# Exploring Personal Recovery in Schizophrenia: The Role of Mentalization

**DOI:** 10.3390/jcm12124090

**Published:** 2023-06-16

**Authors:** Carmen Concerto, Alessandro Rodolico, Ludovico Mineo, Alessia Ciancio, Leonardo Marano, Carla Benedicta Romano, Elisa Vita Scavo, Riccardo Spigarelli, Laura Fusar-Poli, Rosaria Furnari, Antonino Petralia, Maria Salvina Signorelli

**Affiliations:** 1 Psychiatry Unit, Department of Clinical and Experimental Medicine, University of Catania, 95123 Catania, Italy; c.concerto@policlinico.unict.it (C.C.); alessia.ciancio@gmail.com (A.C.); elisavita.scavo@tiscali.it (E.V.S.); petralia@unict.it (A.P.); maria.signorelli@unict.it (M.S.S.); 2 Department of Brain and Behavioral Sciences, University of Pavia, 27100 Pavia, Italy; laura.fusarpoli@unipv.it

**Keywords:** schizophrenia, personal recovery, mentalization, insight

## Abstract

Recovery is a broadly debated concept in the field of psychiatry research and in schizophrenia. Our study aims to understand the correlation between personal recovery from schizophrenia and factors such as mentalization, disability, quality of life, and antipsychotic side effects; Methods: Participants with schizophrenia (according to DSM-5 criteria) were consecutively recruited from the Psychiatry Unit of the University of Catania, Italy. Participants were assessed with the Recovery Assessment Scale (RAS), the Multidimensional Mentalizing Questionnaire (MMQ), the brief version of the WHO Disability Assessment Schedule (WHO-DAS), the EuroQoL-5 dimensions-5 levels, the Insight Orientation Scale (IOS) and the Glasgow Antipsychotic Side Effect Scale (GASS); Results: 81 patients were included. Our findings showed a positive correlation between RAS total scores and MMQ scores, especially in “good mentalizing” subdomains. IOS scores also had a positive association with RAS and MMQ scores. In contrast, poor mentalizing abilities negatively correlated with WHO-DAS 2.0 scores. While antipsychotic side effects influenced functioning, they did not impact perceived recovery. Conclusions: The study’s results identified potential predictors of personal recovery from schizophrenia. These findings could contribute to creating tailored interventions to facilitate the recovery process.

## 1. Introduction

Recently, the concept of recovery in psychiatric disorders, especially schizophrenia, has garnered increasing attention. However, the quest for a comprehensive definition of this construct continues to provoke discussion. Consistent with previous research on patient outcomes, the theorization of recovery in schizophrenia has evolved from a perspective centered on symptom remission (clinical recovery-CR) and functional rehabilitation (functional recovery-FR) to a more holistic and patient-oriented approach emphasizing the personal dimension of the recovery process [1,2].

### 1.1. Personal Recovery

Personal recovery (PR) can be conceptualized as a continuous personal journey of adaptation and growth to overcome the adverse personal and societal consequences associated with any mental disorder [3]. It encompasses various elements, such as spirituality, empowerment, embracing the illness actively, finding hope, restoring a positive identity, creating meaning in life, combating stigma, taking charge of one’s own life, and cultivating supportive relationships [4]. PR concerns the individuals’ perceived capacity to manage mental illness, their sense of purpose, and their confidence in their ability to lead a fulfilling life, irrespective of the disorder’s severity [5]. Thus, “being in recovery” might result from a transformative process involving changes in unique and deeply subjective domains of human experience [6]. Ever since its conceptualization, it has been observed that PR does not necessarily correspond exactly with CR. In a 2018 systematic review and meta-analysis aimed at investigating the relationship between CR and PR, only a significant small to medium association was found between these two distinct concepts of recovery [7]. Therefore, it was emphasized that when treating and evaluating outcomes of patients with schizophrenic spectrum disorders, both CR and PR should be taken into account, but in separate ways. As a complex and multifaceted construct, PR has been operationalized in several different ways with various psychometric tools. In 2011, the CHIME framework for PR was developed providing a coherent and robust structure able to orient research and clinical efforts [8]. The acronym CHIME derives from the five key components of the recovery process, namely “connectedness”, “hope and optimism about the future”, “identity”, “meaning in life”, and “empowerment”. Although there is no consensus on the gold-standard measurement tool for PR, the Recovery Assessment Scale (RAS) by Corrigan et al. [9] is the most commonly referenced in the literature. In a systematic review of PR measures [10], the RAS has shown the broadest array of psychometric properties, appearing to fit well with the CHIME model.

### 1.2. Factors Influencing Personal Recovery

In contrast to the large amount of available evidence [11,12,13,14] on predictive factors of clinical and functional recovery, relatively fewer studies have focused on the potential determinants of its personal counterpart. Among the individual factors affecting PR, older age and a lower level of education have been shown to act as negative predictors [15]. On the other hand, resilience, intended as the inner strength needed to recover, endure, and adapt to life’s pressures, could enhance the perceived recovery in people with a psychiatric disorder, including schizophrenia [16,17]. Regarding the identification of social factors that might impact PR, prior research indicates that aspects such as social support can have a positive effect on the PR trajectory. In contrast, the stigma connected to mental illness is seen to potentially negatively influence the course of PR [17,18,19,20]. Although there is a significant positive correlation observed between FR—interpreted as the individual’s capacity to offset cognitive functioning deficits—and PR, a substantial body of evidence suggests that these domains are not coincidental and display discernible predictive factors along with distinct relationships concerning symptomatology and intrinsic disease characteristics [21,22,23]. In fact, although some studies have reported a positive correlation between PR and neurocognitive function [23,24], a meta-analysis of the determinants of PR found that neurocognition, in general, had no association with PR [25]. While the underlying aspects of PR imply the necessity for a suitable level of reflective functioning, to the best of our knowledge, there have been limited studies investigating the association between mentalization and PR in schizophrenia. Mentalization, often referred to as the theory of mind (ToM), is commonly defined as the “imaginative mental activity through which behavior is interpreted in terms of mental states such as needs, feelings, beliefs and goals” [26]. This cognitive process, which serves as a crucial aspect of social cognition, enables the attribution of mental states, including beliefs, emotions, knowledge, and intentions, by thoroughly employing all available information sources. By integrating and synthesizing this information, it enables the inference of the most appropriate mental state within a given context [27]. Thus, it plays a crucial role in interpersonal relationships through its impact on the interpretation and response to social information by individuals [28]. Importantly, mentalization is not only a cognitive process but also has significant biological underpinnings. Research suggests that mentalization is linked with specific brain structures and networks, notably the right temporoparietal junction, the right middle temporal gyrus, and the left precuneus [29]. It has also been hypothesized that there is a reciprocity between sex hormones and social cognition in schizophrenia, where oxytocin, estrogens, and testosterone could have a role [30], and being exposed to these in fetal life might have an effect on the disorder [31]. An expanding body of literature has documented various deficits in both cognitive and affective facets of mentalizing in individuals diagnosed with psychosis, and the anomalies within this specific metacognitive domain are responsible for a significant portion of the compromised social functioning and poor social outcomes observed [32,33,34]. In our study, we aimed to assess the correlation between PR and independent variables, such as mentalization, disability, quality of life, orientation, tendency toward introspection, and antipsychotic (AP) side effects (SE) in a group of patients with schizophrenia. In particular, we hypothesize that people with schizophrenia who have better mentalization capacity and better orientation and tendency toward introspection might have a stronger subjective experience of recovery.

## 2. Materials and Methods

Participants (81) were consecutively recruited from the Psychiatry Unit of the University of Catania, Italy from September 2021 to April 2022. All patients presented a DSM-5 diagnosis of schizophrenia. Inclusion criteria were: age ≥ 18 years; being an outpatient; diagnosis of schizophrenia based on DSM-5 criteria; absence of positive symptoms at the time of recruitment (defined with a score ≤ 3 on the Positive and Negative Syndrome Scale [PANSS]); the presence of good insight (PANSS g12-lack of judgment and insight ≤ 3); and the ability to read and understand the informed consent documentation. We considered the following as exclusion criteria: concomitant organic diseases; use of psychoactive substances; and other neurological conditions. All patients were taking a second-generation AP as their primary medication. All participants gave their informed consent for data collection. The study was conducted in accordance with the Declaration of Helsinki and was approved by the University of Catania review board. Each participant was asked to fill out a socio-demographic form including socioeconomic variables and psychological variables.

The following questionnaires were administered:

- The Recovery Assessment Scale (RAS) was used to define patients’ perceived recovery. It is a 41-item self-administered tool structured as a Likert scale from 1 (completely disagree) to 5 (completely agree), designed to assess perceived recovery in psychiatric patients. Five factors of recovery can be depicted as Personal Trust and Hope, Willingness to Ask for Help, Goal and Success Orientation, Dependence on Others, and Not being Dominated by Symptoms [4,35].
- The Multidimensional Mentalizing Questionnaire (MMQ) measures the mentalizing processes. It is a 33-item self-rated tool covering different core aspects of mentalization that embraces a multidimensional construct with 4 dimensions: automatic-controlled mentalizing, self/other-oriented mentalization, internal-external mentalizing, and cognitive/affective mentalization. The response format is on a five-point Likert scale from 1 = “Not at all” to 5 = “A great deal”. It can be possible to define scores on positive (reflexivity, ego-strength, and relational attunement) and negative (relational discomfort, distrust, and emotional dyscontrol) subscales as well as an overall MMQ score by summing all the items after having reversed those included in the negative subscales [36].
- The brief version of the WHO Disability Assessment Schedule (WHO-DAS) 2.0 is a 12-item self-rated scale used to measure disability levels in clinical practice [37,38]. All the questions refer to the prior thirty days, asking for the level of difficulty in doing daily activities, ranging from “No difficulty”, equal to 1, to “Extreme or cannot do”, equal to 5. The sum of the items is proportional to the functional impairment. The following six “life areas” can be evaluated: Cognition, Mobility, Self-care, Getting along, Life activities, and Participation.
- The EuroQoL-5 dimensions-5 levels (EQ-5D-5L) was administered to assess quality of life [39]. It is a self-report screening tool consisting of two sections. The first part contains five Likert-level questions regarding movement capacity, self-care, common activities, pain, and anxiety/depression; the second part is a visual analog scale (VAS) in which patients indicate their perceived health ranging from 0 to 100, where higher is better.
- The Insight Orientation Scale (IOS) is a 7-item self-report scale designed to measure a person’s orientation and tendency toward insight, which refers to the understanding or awareness of one’s thoughts, emotions, and behaviors. Each item is rated on a Likert scale ranging from 1 (not at all) to 5 (a great deal), focused on seven core aspects of the construct: level of consciousness, problem solving, restructuring (behavior change), awareness, complexity (abstraction, depth), surprise, and self-reflectiveness (thoughtfulness) [40].
- The Glasgow Antipsychotic Side Effect Scale (GASS) is a 22-item self-rated questionnaire used to assess AP-induced SE. For each item, it is possible to indicate the frequency of the reported SE (Never, Once, A few times, and Every day, scored as 0, 1, 2, and 3, respectively) and then the level of distress that the SE determines (scored from 1 to 10). The first twenty questions refer to the prior week, while the last two questions (on changes in menstrual periods and weight gain) refer to the previous 3 months. The total scale score is given by the sum of the item frequency [41].

### Statistical Analyses

We reported the mean and standard deviation for all variables. If a variable was found to be non-normally distributed, we also included the median and interquartile range in our report. We used the Kolmogorov-Smirnov test to examine the normality distribution of continuous variables. To summarize categorical variables, we displayed both the count and percentage of each category. We calculated a correlation matrix using Spearman correlation coefficients to evaluate the relationship between variable pairs. Since many of the variables did not follow a normal distribution, we utilized Spearman’s correlation rather than Pearson’s correlation for the whole correlation matrix. Multiple univariate regression models were run to investigate the association between multiple variables (age, gender, education, marital status, having children, work, illness duration, hospitalizations, WHO-DAS 2.0 total score, EQ-5D-5L-VAS total score, IOS total score, MMQ total score, and GASS total scores) and the RAS total score and its sub-domains. We reported the model ANOVA *p*-value and its adjusted R^2^ values. We set the alpha level beforehand to 0.05 and implemented the Bonferroni correction by dividing the alpha level by the number of variables evaluated for demographics and psychometric questionnaire items in the correlation matrix.

## 3. Results

Our study included 81 patients diagnosed with schizophrenia, where more than half were male (53) and the rest were female (28). The average age of the participants was 44.2 years (S.D.: 13), and their education level varied: 12% completed elementary school; 36% attended secondary school; 41% completed high school, and 11%completed their graduation. Most of the participants were single (77%) and only a few (19) had children. We found that only a quarter of the participants were employed. More details about illness-related variables and psychometric scores are reported in Table 1.

The correlation matrix in Table 2 suggests a good internal consistency of the individual psychometric instruments; the Cronbach α of the psychometric instrument we used was 0.888 for the WHO-DAS 2.0, 0.851 for the IOS, 0.85 for the MMQ, 0.838 for the GASS, 0.966 for the RAS total score, 0.894 for the Self Trust RAS sub-scale, 0.84 for the Help RAS sub-scale, 0.869 for the Success RAS sub-scale, 0.756 for Other Trust sub-scale and 0.759 for the Not-overwhelmed RAS sub-scale. The IOS score showed a positive correlation with both the RAS total score and the self-trust, help, and success RAS subscales. Similarly, there was a correlation between IOS and MMQ total scores as well as the MMQ reflexivity, ego-strength, and relational attunement sub-dimensions. On the one hand, the RAS total score, as well as several of its sub-scales, have been found to be positively associated with both the MMQ total score and the “good mentalizing” subdomains, including reflexivity, ego-strength, and relational attunement. On the other hand, this correlation is not present with any of the “bad mentalizing” sub-scales. The MMQ subscales related to poor mentalizing abilities, such as relational discomfort, distrust, and emotional dyscontrol, showed a negative correlation with WHO-DAS 2.0 scores. This indicates that a lack of mentalizing ability can hinder an individual’s overall level of functioning. Finally, we checked the impact of AP-SE with GASS, finding it influenced functioning but not perceived recovery.

We conducted multiple univariate regression analyses to examine the relationship between demographic variables and psychometric scales with the RAS total score and its sub-scales (Table 3). All regression models we tested were statistically significant and had high adjusted-R^2^ values that ranged between 0.146 and 0.482. Our findings indicated that the Self-Trust subscale of the RAS was inversely correlated with illness duration, but directly correlated with the IOS and MMQ total scores. Furthermore, the RAS Help subscale was directly associated with the IOS total score, the RAS Success subscale with the MMQ total score, and the RAS Other-Trust subscale with the WHODAS total score. We also found that illness duration was inversely correlated with the Not-Overwhelmed subscale of the RAS. Lastly, the RAS total score was positively correlated with the Insight and Mentalization scales scores.

## 4. Discussion

### 4.1. Findings from the Study

Our study evaluated the associations between a patient-centric viewpoint on recovery and clinical factors in individuals diagnosed with schizophrenia. Results showed that self-reported PR was positively correlated with mentalization. The MMQ subscales “reflexivity”, “ego-strength”, and “relational attunement” were found as the main predictive factors explaining the PR, suggesting that people with schizophrenia who have better mentalization capacity also have a stronger subjective experience of recovery.

### 4.2. Correlation between Mentalization Abilities and Other Variables

In psychosis, the integration of sensory and metacognitive information is commonly impaired [42,43]. A considerable line of research in schizophrenia has highlighted alterations in the mentalization process, and this might explain some aspects of the patients’ social dysfunction and poor social outcomes [34,44]. Deficits in ToM have been observed in psychotic patients with compromised social behavior [32,45,46] and with functional impairment especially in circumstances in which patients needed to cooperate with others [47]. For instance, it has been observed that individuals with psychosis may develop firm beliefs about others’ intentions based just on their physical observable behavior, losing the capacity to consider alternative perspectives [42]. In our study, we adopted a novel tool to investigate the mentalizing processes as a multidimensional construct [48]. The instrument includes domains of “good” and “poor” mentalization. Good mentalizing ability is theorized as the effect of a steadiness between these polarizations that is able to guarantee a flexible use of each dimension according to requirements [49]; meanwhile, mentalizing difficulties are the result of inequalities, poor combination, or unwarranted divergence in the diverse polarities [50]. Our results showed that “good mentalizing” subdomains of the MMQ were positively correlated with the IOS total score, suggesting that the tendency to understand the profound meaning of one’s life events, with the ability to analyze one’s experiences and the ability to manage daily difficulties with a sense of efficacy and realistic confidence, might influence the ability to discern personal desires and protective strategies, along with the capacity to engage with others. There is increasing evidence that functioning difficulties in schizophrenia are linked to social cognition deficits [33]. Mentalization is a key area of social cognition that has been found to be closely linked to general functioning [51,52]. We found that the “poor mentalization” domains, including relational discomfort, distrust, and emotional dyscontrol, had a negative correlation with the WHO-DAS scores. In this case, the scoring of the “poor mentalization” sub-domains of the MMQ has been reversed, meaning that higher values denote better mentalization skills. Concurrently, a higher WHO-DAS score signifies lower functional ability. Given the inverse correlation between these two scales, it follows that enhancement in these “poor mentalization” MMQ domains (interpreted as an improvement due to the inverted values) corresponds to an increase in overall functioning (or a decrease in the WHO-DAS score). In this regard, a previous study by Bellaspì et al. on healthy subjects observed that mentalization was positively associated with self-esteem as well as with general, social, and role functioning, suggesting that good mentalization skills are correlated with global measures of mental health [53]. A recent meta-analysis by Thibaudeau et al. exploring the associations between ToM and different domains of functioning in schizophrenia showed a strong association between mentalization abilities and functioning in areas involving social interactions such as social functioning and productive activities [43]. Most interventions in mental healthcare aim to reduce symptoms and improve functioning [54]. With regard to PR, these interventions may also benefit from focusing on mentalization. Our findings suggest that interventions aimed at improving mentalization ability may enhance PR in individuals with schizophrenia. Indeed, mentalization is commonly targeted to restore mental health, making it a common factor in most psychological treatments. In our study, we also found an advantageous role of IOS on PR, suggesting that a better perception of recovery was correlated with individual awareness of their own thoughts, emotions, and behaviors. It was suggested that self-esteem and hope are important elements of recovery [55]. Certain emotional and personological features may influence PR. Law et al. [56] demonstrated that substantial emotional distress and elevated feelings of hopelessness are unfavorable indicators of PR, whereas a positive sense of self-esteem serves as a favorable one. In the systematic review and meta-analysis by Leendertse et al. [25], the authors investigated factors associated with the PR-scale total scores in people with psychotic disorders. Large positive associations with PR were found for meaning in life, empowerment, and hope. We found that self-trust, help, and success RAS subscales were positively associated with the IOS total scale. This is in line with qualitative studies, which indicated that PR from the point of view of people with psychotic disorders can be defined in terms of faith, hope, agency, and spirituality [57,58]. The impact of disease-related characteristics on PR has also been investigated. Chang et al. (2013), [18] reported that disease duration was a significant predictor of PR in people with psychiatric disabilities and that a better PR status would be exhibited by patients with a longer disease duration of illness. Conversely, we found that the self-trust RAS subscale was inversely correlated with illness duration. It might depend on the highly individual process of PR that poses the patient as the one primarily responsible for his or her individual recovery experience [59]. The functioning measured by the WHO-DAS and antipsychotic side effects, assessed by the GASS, were significantly correlated, indicating that the side effects of antipsychotics can substantially impact an individual’s level of functioning. However, when examining personal recovery, as measured by RAS, we found no significant correlation with functioning. This suggests that an individual’s perceived recovery process is not directly associated with their functional status, highlighting the importance of understanding and addressing these constructs independently in the context of treatment and care for schizophrenia.

### 4.3. Limitations

The current study is not without its limitations. As is common with cross-sectional studies, our research may be subject to temporal bias due to the snapshot nature of data collection. We have not monitored patients longitudinally, which limits our understanding of potential changes and trends over time. We endeavored to create a consistent and homogeneous sample by selecting patients with stable schizophrenia, but this approach may have inadvertently narrowed the scope of our findings, potentially restricting their generalizability to a wider schizophrenia population. A further limitation involves the potential cognitive impairment in our patient group. We did not incorporate measures to account for this factor in our study design. Consequently, any cognitive deficits could have influenced the accuracy of the responses on self-administered scales, potentially introducing bias into our data. Finally, we predominantly relied on self-administered scales without clinician measures for most of our assessments. While this approach has certain advantages, it also introduces the potential for social desirability bias, as patients may respond in ways they perceive as socially acceptable rather than providing entirely accurate responses. These limitations highlight areas for further refinement in future research endeavors.

## 5. Conclusions

Recovery from schizophrenia is a multifaceted process that encompasses numerous elements. The ability to interpret mental states is crucial for understanding human behavior and social interactions. Therapeutic strategies designed to bolster cognitive abilities could potentially boost PR among individuals diagnosed with schizophrenia. The process of mentalization might serve as a mitigating factor in functional outcomes, thereby offering a promising approach to rehabilitation efforts targeting deficient interpersonal functioning.

## Figures and Tables

**Table 1:** Illness-related variables and psychometric scores.

Variables	Mean	Standard Deviation	Median	Inferior IQ	Superior IQ
Illness Duration Years *	13.80	9.68	6	12	20
Hospitalizations *	2.43	3.84	0	1	3
WHODAS Cognition *	3.88	2.15	2	3	5
WHODAS Mobility *	3.99	2.26	2	3	6
WHODAS Selfcare *	2.91	1.78	2	2	3
WHODAS Getting Along *	3.74	2.12	2	3	5
WHODAS Life Activities *	3.89	2.10	2	3	5
WHODAS Participation *	4.30	1.85	3	4	6
WHODAS Total *	22.70	9.52	15	20	28
EQ-5D-5L-VAS	68.62	24.14	50	75	85
RAS Self Trust *	31.53	7.93	28	33	37
RAS Help *	11.65	2.86	10	12	14
RAS Success *	18.74	4.81	16	20	22
RAS Other Trust *	14.72	3.73	13	15	17
RAS Not Overwhelmed *	9.22	3.31	7	9	12
RAS Total *	146.69	31.44	135	150	166
IOS Total	21.25	6.39	-	-	-
MMQ Reflexivity	27.69	8.51	-	-	-
MMQ Ego-strength	15.91	5.83	-	-	-
MMQ Relational attunement	12.84	4.29	-	-	-
MMQ Relational discomfort *	19.64	4.61	18	21	23
MMQ Distrust *	14.01	3.82	12	15	17
MMQ Emotional dyscontrol *	15.17	3.95	13	16	18
MMQ Total	105.27	17.18	-	-	-
GASS Total *	15.07	10.55	6	13	21

RAS: Recovery Assessment Scale; WHODAS: World Health Organization Disability Assessment Scale; EQ-5D-5L-VAS: EuroQol 5-Dimension 5-Level Visual Analog Scale; IOS: Insight Orientation Scale; MMQ: Multidimensional Mentalizing Questionnaire; GASS: Glasgow Antipsychotics Side Effect Scale; non normally distributed variables are marked with “*”.

**Table 2:** Correlation matrix.

		1	2	3	4	5	6	7	8	9	10	11	12	13	14	15	16	17	18	19	20	21	22	23	24	25
Age	1	-																								
Illness Duration Years	2	**0.558**	**-**																							
WHODAS Cognition	3	0.026	−0.059	**-**																						
WHODAS Mobility	4	−0.032	0.040	**0.548**	**-**																					
WHODAS Selfcare	5	0.119	0.123	**0.422**	**0.419**	**-**																				
WHODAS Getting Along	6	−0.027	−0.050	**0.64**	**0.477**	**0.438**	**-**																			
WHODAS Life Activities	7	0.053	−0.025	**0.725**	**0.483**	0.401	**0.604**	**-**																		
WHODAS Participation	8	−0.110	−0.135	**0.514**	**0.477**	0.278	**0.573**	**0.542**	**-**																	
WHODAS Total	9	0.007	0.011	**0.816**	**0.74**	**0.555**	**0.797**	**0.823**	**0.77**	-																
EQ-5D-5L-VAS	10	0.013	−0.147	−0.229	−0.356	−0.159	−0.322	−0.29	−0.352	−0.378	-															
RAS Self Trust	11	−0.033	−0.214	−0.150	−0.204	−0.363	−0.324	−0.215	−0.348	−0.343	**0.57**	**-**														
RAS Help	12	−0.210	−0.171	−0.164	−0.083	−0.287	−0.158	−0.069	−0.089	−0.151	0.305	**0.585**	**-**													
RAS Success	13	0.028	−0.055	−0.045	−0.109	−0.149	−0.173	−0.044	−0.186	−0.159	0.287	**0.672**	**0.564**	**-**												
RAS Other Trust	14	0.052	−0.065	0.160	0.083	0.017	0.069	0.197	0.075	0.135	0.194	**0.467**	**0.43**	**0.488**	**-**											
RAS Not Overwhelmed	15	0.016	−0.175	0.035	−0.139	−0.28	−0.027	−0.034	−0.161	−0.133	0.160	**0.52**	0.222	0.323	0.349	-										
RAS Total	16	−0.005	−0.175	−0.045	−0.179	−0.263	−0.198	−0.107	−0.276	−0.236	**0.468**	**0.904**	**0.661**	**0.771**	**0.644**	**0.625**	**-**									
IOS Total	17	−0.029	−0.051	−0.125	−0.103	−0.255	−0.224	−0.110	−0.039	−0.148	0.297	**0.607**	**0.503**	**0.501**	0.376	0.348	**0.65**	**-**								
MMQ Reflexivity	18	−0.039	−0.084	0.085	0.077	−0.063	0.054	0.070	0.117	0.077	0.101	**0.5**	**0.474**	**0.502**	**0.478**	0.387	**0.621**	**0.714**	**-**							
MMQ Ego-strength	19	−0.099	−0.140	−0.165	−0.23	−0.374	−0.242	−0.256	−0.215	−0.307	**0.423**	**0.684**	0.302	**0.473**	0.242	**0.513**	**0.642**	**0.69**	**0.566**	**-**						
MMQ Relational attunement	20	0.051	−0.016	−0.011	0.083	−0.102	0.030	0.009	0.006	0.027	0.099	0.365	0.291	**0.422**	0.356	0.392	**0.464**	**0.629**	**0.696**	**0.535**	**-**					
MMQ Relational discomfort	21	0.087	0.019	**−0.423**	−0.252	−0.210	**−0.413**	−0.321	−0.369	−0.385	0.257	0.142	0.036	0.061	−0.161	−0.104	0.008	−0.007	−0.277	−0.039	−0.189	**-**				
MMQ Distrust	22	0.073	−0.019	−0.233	−0.195	−0.085	−0.171	−0.186	−0.263	−0.231	0.157	0.004	−0.020	0.073	−0.139	−0.195	−0.027	−0.080	−0.255	−0.120	−0.101	**0.657**	-			
MMQ Emotional dyscontrol	23	0.131	−0.053	−0.4	−0.382	−0.293	**−0.433**	−0.334	**−0.427**	**−0.46**	0.382	0.096	−0.066	−0.083	−0.165	0.070	−0.031	−0.111	−0.331	0.054	−0.196	**0.577**	0.381	-		
MMQ Total	24	0.080	−0.054	−0.278	−0.153	−0.324	−0.269	−0.248	−0.224	−0.286	0.343	**0.602**	**0.425**	**0.527**	0.316	0.379	**0.611**	**0.729**	**0.696**	**0.732**	**0.695**	0.28	0.241	0.234	-	
GASS Total	25	−0.096	0.040	**0.413**	**0.456**	0.222	**0.425**	**0.438**	**0.562**	**0.578**	−0.398	−0.291	−0.025	−0.081	0.086	−0.079	−0.203	−0.036	0.154	−0.152	0.078	−0.395	−0.315	−0.393	−0.182	-

RAS: Recovery Assessment Scale; WHODAS: World Health Organization Disability Assessment Scale; EQ-5D-5L-VAS: EuroQol 5-Dimension 5-Level Visual Analog Scale; IOS: Insight Orientation Scale; MMQ: Multidimensional Mentalizing Questionnaire; GASS: Glasgow Antipsychotics Side Effect Scale; underlined and bold text represent the significant correlations between variables (after Bonferroni correction, *p* < 00016).

**Table 3:** Multiple regression analyses of RAS total and RAS sub-scales scores.

	RAS Self Trust		RAS Help		RAS Success		RAS Other Trust		RAS Not Overwhelmed		RAS Total	
Model Adjusted-R^2^	**0.482**		**0.209**		**0.281**		**0.146**		**0.167**		0.447	
Model ANOVA *p*-value	>0.001		0.005		>0.001		0.029		0.017		<0.001	
	β	t	β	t	β	t	β	t	β	t	β	t
(Constant)		0.851		2.137		0.336		0.175		0.317		0.943
Age	0.108	0.937	−0.097	−0.68	−0.034	−0.251	0.12	0.812	0.2	1.372	0.11	0.928
Gender	0.164	1.947	0.169	1.625	−0.018	−0.179	0.191	1.763	0.195	1.823	0.143	1.642
Education	−0.049	−0.524	0.084	0.727	0.155	1.399	0.044	0.362	−0.121	−1.015	0.038	0.393
Marital Status	−0.134	−1.404	−0.162	−1.372	0.002	0.02	−0.007	−0.059	−0.032	−0.267	−0.09	−0.918
Children	−0.002	−0.017	0.117	0.946	0.125	1.055	0.001	0.011	−0.024	−0.187	0.064	0.616
Work	0.123	1.325	0.163	1.414	0.13	1.185	0.136	1.137	0.179	1.517	0.176	1.829
Illness Duration	**−0.241**	**−2.21**	−0.034	−0.249	−0.02	−0.158	−0.082	−0.586	**−0.316**	**−2.284**	−0.193	−1.708
Hospitalizations	0.146	1.598	0.074	0.651	0.181	1.681	0.118	1.007	0.058	0.496	0.154	1.627
WHODAS Total	0.007	0.065	−0.035	−0.274	0.027	0.222	**0.286**	**2.149**	−0.018	−0.141	0.076	0.713
EQ-5D-5L-VAS	0.156	1.493	0.058	0.448	0.026	0.215	0.016	0.116	−0.164	−1.235	0.051	0.469
IOS Total	**0.286**	**2.232**	**0.397**	**2.512**	0.151	1.005	0.255	1.552	0.17	1.046	**0.35**	**2.646**
MMQ Total	**0.307**	**2.157**	0.063	0.357	**0.395**	**2.359**	0.18	0.987	0.317	1.757	**0.305**	**2.074**
GASS Total	−0.058	−0.554	0.12	0.931	−0.001	−0.008	0.008	0.063	0.019	0.142	−0.019	−0.176

RAS: Recovery Assessment Scale; WHODAS: World Health Organization Disability Assessment Scale; EQ-5D-5L-VAS: EuroQol 5-Dimension 5-Level Visual Analog Scale; IOS: Insight Orientation Scale; MMQ: Multidimensional Mentalizing Questionnaire; GASS: Glasgow Antipsychotics Side effect Scale; *p*-value ≤ 0.05 for the highlighted cells.

## Data Availability

The data presented in this study are available on reasonable request from the corresponding author.
